# Deciphering the immunological interactions: targeting preeclampsia with Hydroxychloroquine’s biological mechanisms

**DOI:** 10.3389/fphar.2024.1298928

**Published:** 2024-02-05

**Authors:** Maja Gajić, Bianca Schröder-Heurich, Karoline Mayer-Pickel

**Affiliations:** ^1^ Department of Obstetrics and Gynecology, Medical University of Graz, Graz, Austria; ^2^ Gynecology Research Unit, Hannover Medical School, Hannover, Germany

**Keywords:** preeclampsia, Hydroxychloroquine, immunity, pathophysiology, hypertension

## Abstract

Preeclampsia (PE) is a complex pregnancy-related disorder characterized by hypertension, followed by organ dysfunction and uteroplacental abnormalities. It remains a major cause of maternal and neonatal morbidity and mortality worldwide. Although the pathophysiology of PE has not been fully elucidated, a two-stage model has been proposed. In this model, a poorly perfused placenta releases various factors into the maternal circulation during the first stage, including pro-inflammatory cytokines, anti-angiogenic factors, and damage-associated molecular patterns into the maternal circulation. In the second stage, these factors lead to a systemic vascular dysfunction with consecutive clinical maternal and/or fetal manifestations. Despite advances in feto-maternal management, effective prophylactic and therapeutic options for PE are still lacking. Since termination of pregnancy is the only curative therapy, regardless of gestational age, new treatment/prophylactic options are urgently needed. Hydroxychloroquine (HCQ) is mainly used to treat malaria as well as certain autoimmune conditions such as systemic lupus and rheumatoid arthritis. The exact mechanism of action of HCQ is not fully understood, but several mechanisms of action have been proposed based on its pharmacological properties. Interestingly, many of them might counteract the proposed processes involved in the development of PE. Therefore, based on a literature review, we aimed to investigate the interrelated biological processes of HCQ and PE and to identify potential molecular targets in these processes.

## 1 Introduction

Preeclampsia (PE) is a hypertensive disorder, occurring solely during pregnancy and is characterized by high blood pressure, proteinuria and/or other organ manifestations such as liver or kidneys (Brown et al., 2018; Author Anonymous, 2019). The exact aetiology of PE is still unknown. However, abnormal placentation in early pregnancy is thought to cause significant physiologic impairment in the mother later on (Zhou et al., 1997). Due to vascular sclerosis and atypical arteriole remodelling in the placenta, there is a gradual onset of placental ischemia (Rana et al., 2019). Consequently, the emergence of anti-angiogenic and pro-inflammatory factors leads to an imbalance that exacerbates competition for binding sites with important angiogenic and growth factors. As a consequence, impaired vessel formation and insufficient vascular adaptation, particularly affecting various organ systems, including cardiovascular, renal, and hepatic systems are subsequent outcomes (Author Anonymous, 2019). One of the main problems in pregnancies complicated by PE, especially at early gestation is the lack of effective, especially causative therapeutic options. Delivery, sometimes unfortunately immediately after diagnosis, is still the main therapy. If untreated, PE might lead to severe maternal complications such as eclampsia, liver rupture, pulmonary oedema, apoplectic insult or renal insufficiency (Jena et al., 2020). The conventional treatment of PE consists of magnesium sulphate for seizure prevention and fetal neuroprotection, as well as antihypertensive agents, either orally or intravenous and delivery (Author Anonymous, 2019). However, preterm delivery due to PE with its consequences is still a huge problem worldwide and prolongation of pregnancy after especially maternal stabilization should therefore be of highest priority.

Several attempts such as supplements and medications have unfortunately not reached its expectations for prevention of PE. Only low-dose aspirin was found to have a modest benefit in reducing the rate of PE. This benefit might only be achieved if low-dose aspirin was started before 16 weeks of gestation (Author Anonymous, 2019). PE is associated with serious and especially long-term maternal and neonatal complications: for the child due to (iatrogenic) preterm delivery as well as fetal growth restriction (FGR) and for the mother an increased risk of cardiovascular morbidity and mortality. (Freeman et al., 2004; Vitoratos et al., 2010; Drost et al., 2012; Groenhof et al., 2017; Cho et al., 2019; Fox et al., 2019). According to the literature, 30% of women with PE develop arterial hypertension and 25% a metabolic syndrome (Hermes et al., 2013; Veerbeek et al., 2015). PE also has an overall negative effect on offspring’s cardiovascular and neurological health (Lu and Hu, 2019). A few studies also have shown the connection between PE and postpartum depression (Mbarak et al., 2019; Caropreso et al., 2020). Poel et al. revealed in their work from 2009, that 1/5 of all women with PE require psychological treatment for several years (Poel et al., 2009). Therefore, the need for new effective medication for PE is high.

Hydroxychloroquine (HCQ) is an antimalarial drug that has been used to treat autoimmune diseases like rheumatoid arthritis (RA) (Kerschbaumer et al., 2020) and systemic lupus erythematosus (SLE) (Fanouriakis et al., 2019). While its exact mechanism of action is not fully understood, it has been shown that HCQ has multiple effects on the systemic and cellular processes. Among them, anti-inflammatory, immunomodulatory and vascular effects could be beneficial for the treatment of PE (Plantone and Koudriavtseva, 2018; Petri et al., 2021). HCQ’s anti-inflammatory effects lie in its capability to reduce the production and release of inflammatory mediators. Since PE is associated with increased systemic inflammation, HCQ-induced reduction of inflammation could potentially have a beneficial effect. Moreover, HCQ immunomodulatory properties are reflected by the inhibition of certain immune cells and suppression of immune responses (Plantone and Koudriavtseva, 2018). PE is thought to involve immune system dysregulation, and modulating immune responses could potentially help in managing the condition. In addition, HCQ has been shown to improve vascular function and reduce endothelial damage (Petri et al., 2021; Dong et al., 2022), which are common aspects of PE. Therefore, it could be beneficial for the management of PE if HCQ could improve these impairments.

This review aims to elucidate the immunological interactions between HCQ and PE focusing on identifying potential molecular targets within these intersecting domains. Based on a literature search we identified well-described mechanisms of action of HCQ in various conditions. Subsequently, we sought to compare these mechanisms with the processes implicated in the development of PE, aiming to identify the most promising targets for future research.

## 2 Pathophysiology of preeclampsia (PE) and its medical approach

PE is a pregnancy-related syndrome (Myatt and Roberts, 2015) that globally affects 4.6% of all pregnancies (Abalos et al., 2013). It is characterized by new onset of hypertension (systolic >140 mmHg and diastolic >90 mmHg) after 20 weeks of gestation, accompanied by proteinuria, dysfunction of maternal organs, haematological abnormalities, and uteroplacental dysfunction (Brown et al., 2018; Author Anonymous, 2019). With over 70,000 maternal deaths and 500,000 fetal deaths every year, PE is one of the leading causes of maternal mortality and morbidity worldwide (Rana et al., 2019). Based on the time of clinical onset of PE symptoms, PE can be classified as early-onset preeclampsia (EO-PE) occurring before 34 weeks of gestation, or late-onset preeclampsia (LO-PE) occurring after 34 weeks of gestation (Tranquilli et al., 2013). However, more than just the difference in time of occurrence divides these two subtypes of PE. EO-PE is less common but is considered the more severe form based on clinical and laboratory outcomes (Wojtowicz et al., 2019). Contrary, LO-PE is usually characterized exclusively by maternal manifestations and is mild to moderate in severity (Lisonkova and Joseph, 2013; Wojtowicz et al., 2019).

Although the underlying mechanisms of PE remain still unclear, several systemic processes have been proposed. Redman postulated a two-stage model of the disease (Redman, 1991) which has been updated in the past years and has been revised by Staff (Staff, 2019). The latest revision of this model takes into account maternal factors and differences between EO-PE and LO-PE (Staff, 2019). In the newest revision of Redman´s model, stage 1 is characterized by placental dysfunction, while stage 2 represents the clinical manifestation in the mother, both mediated by syncytiotrophoblast stress (Staff, 2019). The pathophysiological process of EO-PE is thought to initiate abnormal trophoblast invasion early in pregnancy, leading to inadequate remodelling of spiral arteries and poor placental perfusion with consecutive oxidative stress and dysfunction of the syncytiotrophoblast. Huppertz *et al.* have shown that in PE syncytiotrophoblast shedding occurs rather in a necrotic than in an apoptotic manner (Huppertz et al., 2003). As a result, syncytiotrophoblasts release trophoblast debris containing damage-associated molecular patterns (DAMPs), pro-inflammatory cytokines and anti-angiogenic factors.

In contrast, LO-PE occurs in term or post-term pregnancies and is characterized by the decline in placental function resulting in increased compression of the intervillous space and hypoxia, which in turn leads to various clinical manifestations of placental dysfunction such as PE, fetal growth restriction, and even stillbirth (Staff, 2019). Both pathways are known to be due to syncytiotrophoblast stress and have similar maternal consequences (Staff, 2019). However, they are triggered by different causes at different times during pregnancy.

Currently, there is still no effective medication for curing PE, and the only curative therapy for PE is the termination of pregnancy. However, according to clinical practice guidelines, the decision for immediate delivery depends on gestational age and maternal and fetal status (Brown et al., 2018). In cases of LO-PE, delivery is advised (Brown et al., 2018; Author Anonymous, 2019). However, for those with PE onset occurring before 34th weeks of gestation (EO-PE), a strategy of expectant management is typically recommended (Brown et al., 2018; Author Anonymous, 2019). Different medications like low-dose aspirin (Phipps et al., 2019), statins (Smith and Costantine, 2022) and calcium (Burton et al., 2019) have been proposed for prevention or even stabilization of PE. However, they are just partially effective in reducing the risk of PE or prolongation of pregnancy.

Only low-dose aspirin was found to have a modest benefit in reducing the rate of PE; this benefit might be achieved if low-dose aspirin was started before 16 weeks of gestation. However, this does not apply to LO-PE. The molecular mechanism of low-dose aspirin in PE is nicely reviewed by Walsh and Straus (Walsh and Strauss, 2021). By blocking its main target cyclooxygenase enzymes, low-dose aspirin specifically reduces the production of thromboxane in maternal platelets without impacting the synthesis of prostacyclin. Furthermore, it seems to similarly target and reduce thromboxane production in the placenta, along with mitigating oxidative stress within the placenta (Gimenez-Bastida et al., 2019; Walsh and Strauss, 2021). However, it has been postulated that the use of aspirin during pregnancy might be associated with increased postpartum bleeding and postpartum hematoma (Hastie et al., 2021; Jiang et al., 2023). Therefore, “Lifestyle modification”, such as optimizing pre-pregnancy weight might be a promising approach (Author Anynomus, 2018; Roberge et al., 2018).

Other candidates are statins, specifically pravastatin. Although preclinical studies (Brownfoot et al., 2015; Brownfoot et al., 2016) and clinical studies with women at high risk of PE (Costantine et al., 2016) showed that pravastatin increases Placental Growing Factor (PlGF) while decreasing Soluble Vascular Endothelial Growth Factor Receptor 1 (sFlt-1) and soluble endoglin (sENG), clinical trials which included EO-PE patients did not observe the same (Ahmed et al., 2020).

High-dose calcium reduces the risk of PE in women destined for the development of PE, while low‐dose calcium did not have a clear effect (Hofmeyr et al., 2018). The need for new treatment options is therefore high. Considering the diverse underlying mechanisms and clinical presentations of PE, it is crucial to acknowledge that a single drug cannot universally address all cases. The heterogeneity of this condition necessitates tailored approaches for effective treatment.

### 2.1 Innate immune system dysregulation in PE

As stated before, in EO-PE necrotic syncytiotrophoblasts release a mixture of molecules, including DAMPs, into the maternal circulation (Staff, 2019). DAMPs are endogenous molecules released from damaged or stressed cells in the body, such as DNA and RNA fragments, ATP, uric acid, heat shock proteins, etc (Piccinini and Midwood, 2010). Similar to pathogen-associated molecular patterns, they can be recognized by pattern recognition receptors on immune cells, such as Toll-like receptors (TLRs). Depending on the type of TLR it can be either present on the cellular membrane or intracellularly inside of endosome.

The main function of endosomal TLRs is to recognize free amino acids derived from bacteria and viruses or necrotic cells. TLR3 bind double-strand RNA (dsRNA) and signals through a MyD88-independent pathway. This way it activates interferon regulatory factor 3 (IRF-3) and increases interferons production in cells (Piccinini and Midwood, 2010). However, in the later phase, it can also activate the nuclear factor kappa-light-chain-enhancer of activated B cells (NF-κB) transcriptional factors and increase pro-inflammatory cytokines (Piccinini and Midwood, 2010). In contrast, TLR7/8 and TLR9 recognise single-strand RNA (ssRNA) and hypomethylated 2′-deoxyribo (cytidine–phosphate–guanosine) (CpG)-rich DNA motifs, respectively (Piccinini and Midwood, 2010). This signal goes through the MyD88-dependent pathway leading to the release of NF-κB translocating to the nucleus, where it triggers the transcription of pro-inflammatory genes, such as cytokines and chemokines (Piccinini and Midwood, 2010).

In the context of PE, TLR3, 7/8 and 9 are found to be increased in PE placentas (Pineda et al., 2011; Chatterjee et al., 2012; He et al., 2018; Ozeki et al., 2019; Gierman et al., 2021). Moreover, the expression of TLR9 co-localised with TLR3 in placental compartments: the trophoblast (syncytium) covering the entire cellular area and in the stromal vascular endothelium (Pineda et al., 2011). In addition to its increased presence in the placenta, TLR9 and 3 expression is also increased in peripheral blood mononuclear cells (PBMCs) in maternal circulation (Liao et al., 2009; Panda et al., 2012).

Regarding ligands of endosomal TLRs, their amount and composition are investigated in the context of PE, too. The presence of cell-free nucleic acids in maternal circulation is not limited to cell-free fetal DNA (cffDNA) but also includes mitochondrial DNA, messenger RNA, microRNA and long non-coding RNA species (Hahn et al., 2014). However, cffDNA has been the most studied one.

Compared to maternal DNA, cffDNA is hypo-methylated (Schroeder et al., 2013) making it recognizable by TLR9. In healthy pregnancies, the concentration of cffDNA in maternal circulation increases with gestational age due to apoptosis of syncytium (Halperin et al., 2000). As previously described, during PE shedding of syncytium is more in a necrotic manner, which is due to placental oxidative stress (Huppertz et al., 2003). Consistent with this, the increase of cffDNA in the maternal circulation is also associated with oxidative stress in the placenta (Tjoa et al., 2006). Therefore, numerous studies have likely detected higher cffDNA levels in pregnancies with PE compared with those without complications (Amash et al., 2012; Yu et al., 2013; Dong and Yin, 2014; Ma et al., 2019; Ozeki et al., 2019) suggesting a possible link between PE and elevated cffDNA levels. In addition, cffDNA levels also correlate with disease severity (Zhong et al., 2001).

Among the different types of cell-free RNA, composition is more important than quantity. Transcriptomic analyses show two different RNA profiles in normotensive and PE patients (Moufarrej et al., 2022; Zhou et al., 2023). However, it would be very plausible that dsRNA and ssRNA are increased in the maternal circulation and can activate TLRs in surrounding cells. In addition, it is likely that high concentrations of nucleic acids in the maternal circulation act as a danger signal to the mother (Scharfe-Nugent et al., 2012).

The connection between endosomal TLRs activation by their ligands and PE pathophysiology is supported by multiple *in vitro* and *in vivo* experiments (Nakada et al., 2009; Tinsley et al., 2009; Chatterjee et al., 2012; Scharfe-Nugent et al., 2012; Goulopoulou et al., 2016; Conka et al., 2017; He et al., 2018; Ozeki et al., 2019). Scharfe-Nugent *et al.* showed that, PBMCs from pregnant women incubated with cffDNA lead to activation of the pro-inflammatory transcriptional factor NF-κB and the production of interleukin (IL)-6 (Scharfe-Nugent et al., 2012). Moreover, Nakada and colleagues observed that after stimulation of HTR-8/SVneo, trophoblast cell line model, with TLR3 specific ligand there is an activation of both NF-κB and IRF3 pathways, leading to elevated inflammatory mediators’ expression and - more interestingly - PE-related anti-angiogenic factor sFlt-1 (Nakada et al., 2009). In addition, it has been shown that activation of TLR9 with cffDNA causes syncytial inflammation (Scharfe-Nugent et al., 2012), promoting both IL-6 and IL-8 secretion by human umbilical vein endothelial cells (HUVECs) (Ozeki et al., 2019) and a sFlt-1 increase while decreasing vascular endothelial growth factor A (VEGFA) in mouse placenta and HTR-8/SVneo trophoblast cell line (He et al., 2018). *In vivo* experiments in rodents have shown, with one exception (Conka et al., 2017), that cffDNA as well as TLR3 and TLR7/8 ligands can trigger fetal loss and induce PE-like conditions (Tinsley et al., 2009; Chatterjee et al., 2012; Scharfe-Nugent et al., 2012; Goulopoulou et al., 2016; He et al., 2018).

The identified findings emphasize the significance of nucleic acids (especially cffDNA) in the development of PE, indicating their potential role as diagnostic markers for early detection of PE (Sekizawa et al., 2003; Karapetian et al., 2021; Moufarrej et al., 2022; Zhou et al., 2023). Moreover, these findings suggest that endosomal TLRs activation could serve as a potential target for the treatment of PE (Pineda et al., 2011; Panda et al., 2012; Scharfe-Nugent et al., 2012).

Another well-characterized TLR is TLR4, which is responsible for the recognition of cell wall components from Gram-negative bacteria (Asea et al., 2002). From the DAMPs, it is responsible for the recognition of heat shock proteins (HSP) (Banerjee et al., 2021), High-Mobility Group Box 1 protein (HMGB1) and uric acid. TLR4 expression was found to be higher in PE (Chen et al., 2015), especially in EO-PE (Kim et al., 2005; Xie et al., 2010; Pineda et al., 2011; Zhang and Yang, 2012; Nizyaeva et al., 2019; Zhao et al., 2019; Romao-Veiga et al., 2020). An increased expression in both first and third-trimester placenta (Zhang and Yang, 2012), interstitial trophoblasts (Kim et al., 2005), syncytiotrophoblast and endothelial cells (Nizyaeva et al., 2019; Zhao et al., 2019) was detectable where it co-localises with TLR2 (Pineda et al., 2011). In addition to placental expression, baseline expression of TLR4 was found to be significantly elevated in monocytes (Chen et al., 2015; Romao-Veiga et al., 2020) and neutrophils (Xie et al., 2010).

Among the DAMPs, HSPs have varying roles in PE. For instance, cell-free HSP70s are increased in the sera of PE patients (Fukushima et al., 2005; Molvarec et al., 2006; Hromadnikova et al., 2016), particularly in EO-PE cases. (Jirecek et al., 2002; Peracoli et al., 2013; Alvarez-Cabrera et al., 2018; Romao-Veiga et al., 2020). They are believed to contribute to the systemic inflammatory response in PE (Asea et al., 2002; Piccinini and Midwood, 2010; Romao-Veiga et al., 2022). Contrary, HSP70 has been found to be increased in the placental tissue (Padmini et al., 2012) but here it has a rather protective function. Moreover, data regarding HSP90 and HSP60 in PE is more diverse. While HSP90 was found to be increased in the sera of PE patients during the third trimester (Guzel et al., 2019), its concentration in endothelial cells isolated from PE placentas showed contrary results (Gu et al., 2006; Padmini et al., 2012). Some studies showed increased levels of HSP60 in PE patients’ sera (Alvarez-Cabrera et al., 2018), while others did not observe any difference (Peracoli et al., 2013).

HMGB1 is a molecule with a dual role. It acts as a transcription factor, when inside cells, while upon release from immune or necrotic cells, it works like a danger signal (Nadeau-Vallee et al., 2016). HMGB1 has been found to be increased in the placenta and maternal circulation in cases of PE (Wang et al., 2011; Chen et al., 2016; Hu et al., 2018). Its presence in syncytiotrophoblasts is particularly associated with severe and EO-PE, (Chen et al., 2016), being released into the maternal circulation following hypoxia-induced necrosis (Hu et al., 2018). The release of HMGB1 from hypoxic trophoblast has been linked to increased permeability (Jiang et al., 2014) and secretion of pro-thrombotic microparticles in HUVECs (Hu et al., 2018) through experiments done on primary human villous trophoblasts, primary mouse trophoblasts and JEG-3 trophoblast cell line. Although, HMGB1 can be recognized by multiple receptors on various cell types (Tang et al., 2011), in HUVECs, HMGB1 induced endothelial permeability through the TLR4– caveolin-1 pathway (Jiang et al., 2014). This pathway may play a role in the clinical features of PE, including general oedema and proteinuria. Furthermore, it has been implicated that HMGB1 could cause increasing reactive oxygen species (ROS) levels as well as IL-6, IL-8 and monocyte chemoattractant protein-1 secretion from Sw.71 human trophoblast cell line (Shirasuna et al., 2016). Additionally, HMGB1 derived from necrotic cells serves as a crucial activator of dendritic cells (DCs) (Tang et al., 2011).

## 3 Hydroxychloroquine (HCQ)

HCQ is a less toxic hydroxylated analogue of an anti-malaria drug chloroquine (Gies et al., 2020). Nowadays, HCQ is widely used in the prevention and treatment of different autoimmune diseases, such as SLE (Fanouriakis et al., 2019), RA (Kerschbaumer et al., 2020), antiphospholipid syndrome (APS) (Tektonidou et al., 2019), and Sjögren’s syndrome (Brito-Zeron et al., 2014; Demarchi et al., 2017; Wang et al., 2017). HCQ has been shown to cross the placental barrier (Costedoat-Chalumeau et al., 2002), and its safety during pregnancy has been already confirmed in a few studies (Abarientos et al., 2011; Kaplan et al., 2016; Clowse et al., 2022). HCQ is associated with higher gestational age at delivery, suggesting its positive effect on the prolongation of pregnancy (Sciascia et al., 2016; Kroese et al., 2017; Gerde et al., 2021) and an increase in live births in patients with APS (Mekinian et al., 2015; Sciascia et al., 2016; Gerde et al., 2021). Some retrospective studies and meta-analyses associated HCQ with a lower risk of PE in SLE (Do et al., 2020; Hu et al., 2023; Phillips et al., 2023) and in APS patients (Sciascia et al., 2016; Latino et al., 2020) who continued to take it during pregnancy.

The most well-known role of HCQ is the alkalization of lysosomes (Derendorf, 2020). In its chemical structure, HCQ has a basic side chain that makes it a weak base. HCQ easily passes through phospholipid membranes and enters the cell and cell compartments. Because of its basic chain, it accumulates in a lysosomal compartment (Gies et al., 2020). There, it alkylates lysosomal pH, which affects several cellular processes (Ponticelli and Moroni, 2017) such as activation of TLRs, antigen processing, major histocompatibility complex class II and autophagy (Mauthe et al., 2018).

Overall, HCQ´s mechanism of action involves inhibiting TLR-mediated cell activation, reducing cytokine production, inhibiting antigen presentation, and modulation of T- and B-cell activation and differentiation. Since PE is a multifactorial disease, there are multiple sources and triggers of cytokine storms. Some of the most important triggers are DAMPs and their receptors on different cells are the first connection between HCQ and PE.

### 3.1 HCQ effect on toll-like receptors and PE

There are two proposed mechanisms for how HCQ inhibits the activation of TLR3, 7, 8 and 9 (Abarientos et al., 2011). The first is the alkalization of lysosomes. An increase in pH prevents proteolytic cleavage of TLR which is required for TLR3, 7 and 9 activation (Ewald et al., 2008; Ewald et al., 2011). The second mechanism involves direct binding to DNA and RNA via electrostatic forces, hydrogen bonds, and van der Waals forces. Thus, HCQ prevents the binding of nucleic acids to TLR receptors present in the endosome (Kuznik et al., 2011). Regardless of the mechanism of action, HCQ prevents the activation of endosomal TLRs in both cases. Therefore, there is no activation of downstream molecules and no increase in inflammation.

**FIGURE 1:**
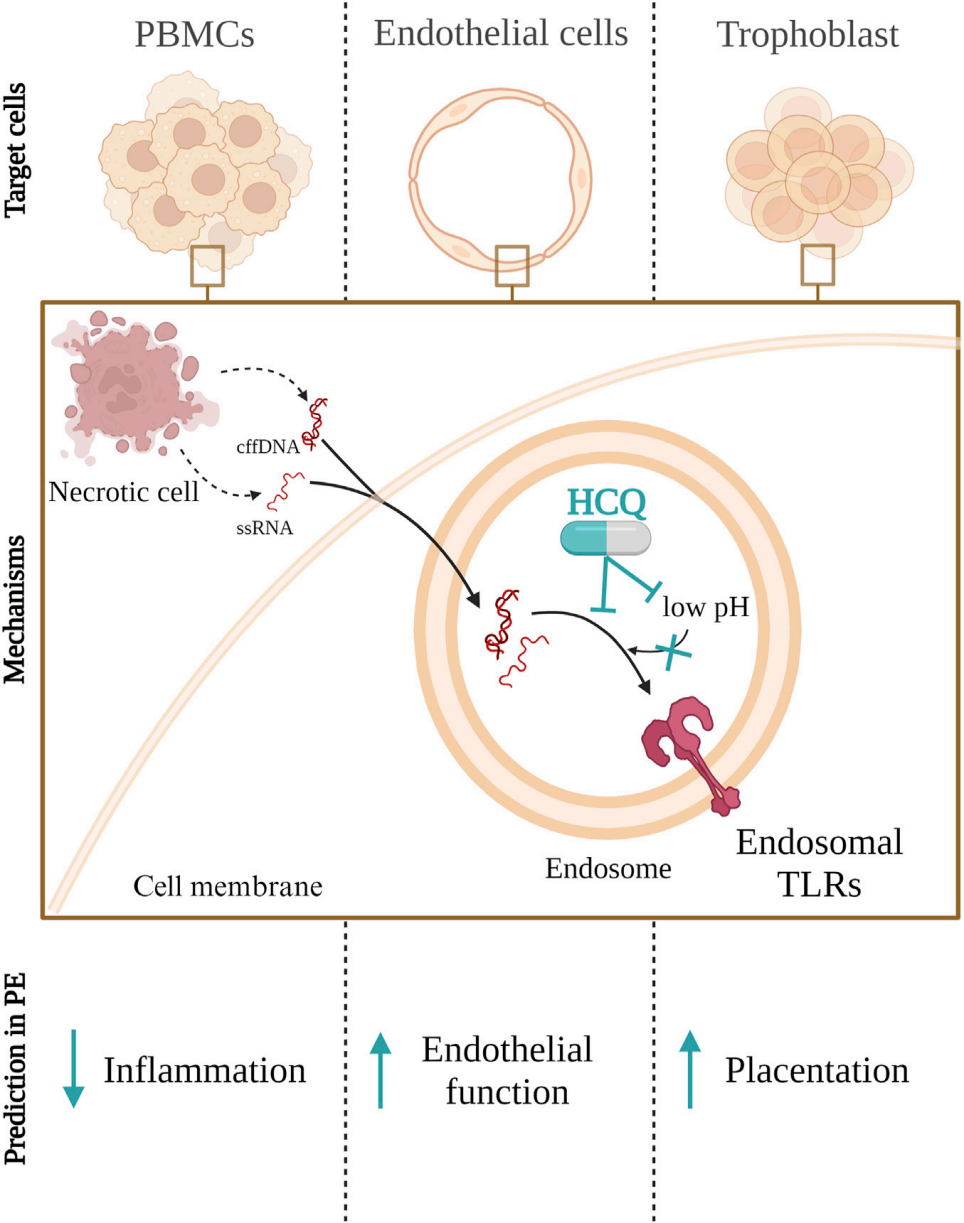
Hydroxychloroquine (HCQ) inhibits the activation of Toll like receptor (TLR) 3, 7, 8, and 9 through two mechanisms: by increasing lysosomal pH, preventing proteolytic cleavage required for TLR activation, and/or by directly binding to DNA and RNA, thus preventing their interaction with TLR receptors in endosomes. This inhibition of endosomal TLR activation in peripheral blood mononuclear cells (PMBCs), endothelial or trophoblasts cells could lead to a reduction in inflammation as well as improve endothelial function and placentation in peeclamptic patients. cffDNA–cell free fetal DNA; ssRNA–single strand RNA.

Considering the HCQ mechanisms of action, we might expect HCQ to block either the binding of DAMPs specific for endosomal TLRs or the activation of endosomal TLRs in PE (Figure 1). This may lead downstream to a decrease of IL-6 and IL-8 production by PBMCs, endothelial cells and trophoblast (Scharfe-Nugent et al., 2012; Ozeki et al., 2019). Further, it is plausible that HCQ could also decrease sFLT-1 and increase VEGFA in maternal circulation (He et al., 2018), which would improve endothelial function and vascularization. However, in a study on term placental explants, HCQ did not significantly decrease the secretion of pro-inflammatory or transcription of angiogenic molecules, induced by TLR7 and TLR9 activation (Scott et al., 2019). The exception was IL-10 secretion, where HCQ increased basal secretion by term placenta explants as well as reversed the negative effect of TLR7 agonist on term placenta explants IL-10 production (Scott et al., 2019). HCQ could most likely improve migration and invasion in early placentation through a TLR9-dependent manner, as this concept was tested in trophoblast cell lines and placental explants (He et al., 2018; Scott et al., 2019; Leon-Martinez et al., 2023).

In contrast to intracellular TLR receptors, HCQ does not affect the activation of TLR4 but reduces the total mRNA and protein amount of TLR4 in cells and it may affect cytokine production (Molvarec et al., 2006; Romao-Veiga et al., 2020). The mechanism of action, in this case, is different from that for endosomal TLRs. HCQ’s impact on TLR4 and HMGB1 modulation has shown promising results (Hu J. et al., 2022; Marchetti et al., 2014; Yang et al., 2013; van den Borne et al., 1997). Studies conducted on microglia and BeWo cells have indicated that HCQ administration inhibits inflammatory responses and affects the expression of TLR4, potentially inhibiting the TLR4/NF-κB signalling pathway (Yang et al., 2013; Marchetti et al., 2014; Hu J. et al., 2022). Additionally, it has been demonstrated that HCQ effectively inhibits the release and pro-inflammatory function of HMGB1 in several *in vitro* cell types and in animal models of endotoxemia and sepsis (Yang et al., 2013).

Moreover, chloroquine (CQ), the precursor for HCQ, is found to decrease the production of Tumor necrosis factor-alpha (TNF-α), IL-1 beta (β) and IL-6 from lipopolysaccharide (LPS)-stimulated human monocytes (Jang et al., 2006). Here it was described that CQ blocks the conversion of cell-associated TNF-α precursor to mature soluble protein, whereas it reduces the levels of IL-1β and IL-6 mRNA, at least in part, by decreasing their stability and by a pH-dependent mechanism. Moreover, LPS-induced IL-6 and TNF-α production were inhibited by both CQ and HCQ in PBMC (van den Borne et al., 1997). Contrary, other authors reported that HCQ was not able to prevent TNF-α secretion triggered by TLR4 activation (Sacre et al., 2012; Alves et al., 2017).

**FIGURE 2:**
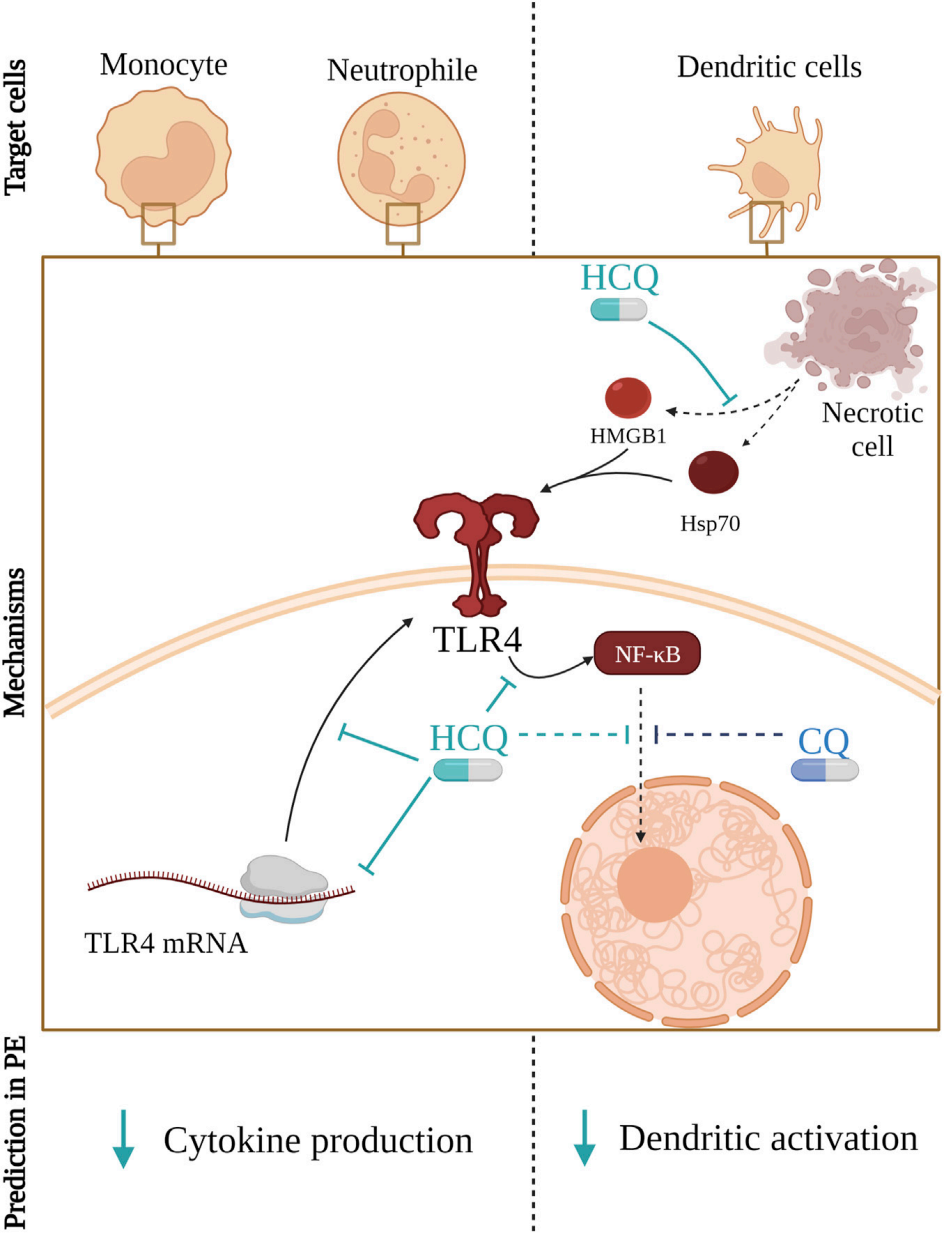
Hydroxychloroquine (HCQ) reduces the amount of available TLR4 on the cell membrane by affecting its mRNA transcription and translation. Furthermore, it blocks signal transduction from TLR4 to NF-κB and, same as Chloroquine (CQ), it prevents NF-κB transport from cytoplasm to the nucleus. Consequentially, that leads to reduced secretion of pro-inflammatory cytokines by monocytes and neutrophils. Furthermore, HCQ mitigates the release of HMGB1 from various cells, thereby preventing TLR4 activation potentially improving endothelial dysfunction and reducing the risk of thrombosis.

The mechanism shown in Figure 2 described in other conditions could also apply to PE. Therefore, HCQ could lead to improvement of PE symptoms by decreasing the total amount of TLR4 might be expected. That would consequentially lead to reduce the secretion of pro-inflammatory cytokines by monocytes and neutrophils. In addition, HCQ could decrease the release of HMGB1 from multiple cells in PE, hence TLR4 activation would not occur. Therefore, HCQ could also improve endothelial dysfunction and thrombosis as well as reduce activation of DCs.

### 3.2 Targeting dysregulated complement in PE with HCQ

When overlapping the information, it is evident that both PE and certain autoimmune conditions, where HCQ is effective, involve the dysregulation of the complement system. The ability of HCQ to inhibit complement activation suggests its potential utility in managing conditions like PE, especially considering its protective effects in related pregnancy complications. However, when we focus on just research that shows the exact components of complement that are targets of HCQ and compare them to components of complement that are different in PE, the overlap is missing.

In the realm of PE, excessive activation of the complement system is widely recognized as a contributing factor to its development, a point underscored by multiple researchers (Alrahmani and Willrich, 2018; Burwick and Feinberg, 2022). In a study that involved 701 pregnant women, Lynch et al. found that elevated levels of the complement activation product Bb in early pregnancy were linked to an elevated risk of PE (Lynch et al., 2008). In contrast, Derzsy et al. did not observe any difference in circulating levels of Bb between healthy and PE women (Derzsy et al., 2010). Multiple authors found an increased presence of C4d in PE placental tissue (Buurma et al., 2012; Yonekura Collier et al., 2019). Moreover, they also found a strong association between these two molecules with and well-known anti-angiogenic factor, sFlt-1 (Yonekura Collier et al., 2019). In serum from pregnant women C4d, C3a, and SC5b9 were higher (Derzsy et al., 2010; Denny et al., 2013; Guseh et al., 2015; Burwick et al., 2018; He et al., 2020).

Conversely, HCQ’s role in inhibiting complement system activation, particularly in pregnancy-related complications and autoimmune diseases, has been explored but lacks specificity in the context of PE. A study by Bertolaccini et al. found that HCQ prevents placental ischemia and abnormal foetal brain development in mice models for antiphospholipid syndrome by inhibiting complement activation (Bertolaccini et al., 2016). In these mice, C3a and C5a levels strongly decreased after HCQ treatment (Bertolaccini et al., 2016). However, we do not know if the reason for that is decreased antiphospholipid antibody binding or if HCQ additionally affects C3 and C convertase. In a study done on syncytiotrophoblast, HCQ impaired complement-dependent antigen-antibody reactions and showed potential protection in APS (Wu et al., 2011; Mekinian et al., 2015). Therefore, the HCQ effect on complement is connected to APS antibodies (Wu et al., 2011). In addition, it has been observed that HCQ therapy in Japanese SLE patients even increases C3 and C4 (Ikeda et al., 2019). However, it was not clarified how. It could be through the inactivation of convertases or by an increase in the production of these molecules. If it is further, that would not be favourable for PE patients. In summary, while HCQ shows promise in modulating the complement system, its specific effects on the complement components involved in PE require further investigation. Direct studies focusing on HCQ’s application in PE are essential to determine its effectiveness and safety in this particular context.

## 4 Adaptive immune system in PE and HCQ immunomodulatory properties

The activation of innate immunity cells and antigen-presenting cells detected in PE results in the production of inflammatory cytokines and activation of cells of adaptive immunity, leading to increased inflammation. Miller *et al.* have provided a comprehensive review of available literature about the role of each immune cell type response in healthy and PE pregnancies (Miller et al., 2022) focusing on T cell subtypes in placental and decidua (Miller et al., 2022). Thus, our focus is on subtypes of T helper cells in maternal circulation, their contribution to cytokine storm and potential interaction with HCQ.

### 4.1 Cytokine effects on Th cell polarization in PE

CD4^+^ T lymphocytes orchestrate adaptive humoral and cell-mediated immune responses (Honey and Rudensky, 2003). Their activation depends on three steps: recognition of antigenic fragments on MHC class II molecules on APC cells, co-stimulating by APC cells and inflammatory cytokines produced by the APC or other cells at the site of T cell activation. This leads to the proliferation and differentiation of activated CD4^+^ T cells (Magombedze et al., 2013). Depending on the particular cytokine milieu, naïve CD4^+^ T lymphocytes can differentiate into five major subsets: T helper (Th) 1, Th2, Th17, T regulatory (Treg), and follicular Th cells (Magombedze et al., 2013; Zhu and Zhu, 2020). Multiple studies have shown that the balance between Th1, Th2, Th17 and Treg lymphocytes in the circulation of PE patients is disturbed (Saito et al., 1999; Santner-Nanan et al., 2009; Darmochwal-Kolarz et al., 2012; Ribeiro et al., 2017; Zhang et al., 2017; Salazar Garcia et al., 2018; Eghbal-Fard et al., 2019; Romao-Veiga et al., 2022). Salazar Gracia and colleagues reported that elevated Th17/Treg cell ratios in peripheral blood happen in early pregnancy and it associated with PE, suggesting a potential predictive marker (Salazar Garcia et al., 2018). Others have shown that the severity of PE is related to the change in Th17/Treg ratio (Ribeiro et al., 2017; Zhang et al., 2017). Multiple authors proposed that an imbalance between Treg cell and Th17 cell differentiation may explain PE pathogenesis (Santner-Nanan et al., 2009; Saito, 2010; Salazar Garcia et al., 2018; Eghbal-Fard et al., 2019). He *et al.* postulated the hypothesis that sENG produced by endothelial cells and syncytiotrophoblasts may disturb Treg cell differentiation (He et al., 2018). Therefore, drugs with immunomodulatory properties which would be able to turn over Th balance in favour of Treg and Th2 would be good candidate for PE treatment.

Cytokines are important for the differentiation of Th cells. However, each Th cell type is also secreting a specific cytokine profile that promotes or blocks differentiation of other Th lymphocytes, leading to a complex feedback loop and an overall cytokine storm. Th1 and Th17 are considered more pro-inflammatory subtypes because they are major producers of TNF-α and INF-γ or IL-17 as well as IL-21 and IL-22, respectively. Contrary, Th2 secreting IL-4, IL-5, IL-6 and IL-13 and Treg secreting TGF-β and IL-10 are considered more anti-inflammatory (Figueiredo and Schumacher, 2016). High levels of pro-inflammatory cytokines are characteristic of early implantation and parturition (Mor et al., 2011; Rinaldi et al., 2017). However, their persistent state leads to pregnancy-related complications (Saini et al., 2011). In women with PE, the cytokine profiles in peripheral blood are mainly Th1 and Th17 dominant (Arriaga-Pizano et al., 2005).

TNF-α is a multifunctional Th1 cytokine and one of the most important inflammatory cytokines. While some studies did not report a difference in TNF-α concentration between maternal sera/plasma from normal and PE pregnancies (Valencia-Ortega et al., 2019), multiple other studies revealed significantly higher TNF-α levels (Serin et al., 2002; Pinheiro et al., 2015; Salazar Garcia et al., 2018; Aggarwal et al., 2019). In addition, Ribeiro *et al.* showed that this increase is more prominent in EO-PE than in LO-PE (Ribeiro et al., 2017). The increase of systemic TNF-α levels during pregnancy, besides PE, is associated with miscarriages, late fetal losses, and preterm birth (Dai et al., 2022). It also inhibits trophoblast-derived JEG-3 cell line integration into endothelial cellular networks *in vitro* co-culture with uterine-derived endothelial cells (Xu et al., 2011). Furthermore, it inhibited EVT invasion (Bauer et al., 2004; Renaud et al., 2005; Otun et al., 2011), implicating possible effects on first-trimester trophoblast invasion and remodelling of the spiral artery. In addition, it has been shown that the challenge of first-trimester placenta explants with TNF-α would drastically increase the shed of syncytiotrophoblasts (Chen et al., 2010) suggesting a TNF-α role in PE origin. PMBCs from PE patients also produce a higher amount of TNF-α compared to PMBCs from healthy pregnant women (Romao-Veiga et al., 2022). Thus, TNF-α acts as one major mediator in the pathogenesis of severe PE (Alijotas-Reig et al., 2017).

The second Th1-produced cytokine is IFN-γ (Figueiredo and Schumacher, 2016). Its circulating (Arriaga-Pizano et al., 2005; Yang et al., 2014; Pinheiro et al., 2015; Deng et al., 2020) and placenta tissue (Sheibak et al., 2020; Liu et al., 2021) levels are increased in PE patients. It has been proposed that IFN-γ could be responsible for the shallow invasion of extravillous trophoblasts since IFN-γ reduced the invasion of the HTR-8/SVneo trophoblast cell line (Liu et al., 2021; Nurzadeh et al., 2023).

Similar to TNF-α, data on IL-6 concentration in the maternal circulation are inconsistent. Various studies demonstrated increased (Takacs et al., 2003; Xiao et al., 2012; Pinheiro et al., 2015; Li et al., 2016; Aggarwal et al., 2019; Ma et al., 2019; Valencia-Ortega et al., 2019; Gencheva et al., 2022; Kadife et al., 2022) rather than unchanged (Borekci et al., 2007; Salazar Garcia et al., 2018) IL-6 concentration in maternal sera and plasma. Xiao *et al.* found a connection between IL-6 and the severity of PE (Xiao et al., 2012). However, Gencheva *et al.* did not observe the same connection (Gencheva et al., 2022). In addition, Takacs *et al.* show that HUVECs incubated with plasma from PE patients secrete more IL-6 (Takacs et al., 2003).

Data regarding IL-1β in PE patients is also varied. While some authors did not observe any difference between maternal sera concentration of IL-1β (Valencia-Ortega et al., 2019; Deng et al., 2020), others found significantly increased concentrations in both maternal sera and in placentas of women with PE (Amash et al., 2012; Dong and Yin, 2014; Ma et al., 2019).

While IL-6 and IL-1β are important for Th17 differentiation, IL-17 is produced by them and plays a well-established role in driving various inflammatory pathways. It is also increased in sera from women with PE (Saito, 2010; Szarka et al., 2010; Molvarec et al., 2015; Eghbal-Fard et al., 2019; El Shahaway et al., 2019; Deng et al., 2020). Its dynamic role involves recruiting and activating neutrophils, which stands as a distinctive characteristic of the inflammatory response observed in PE (El Shahaway et al., 2019). In a pregnant rodent treated with TLR3 and TLR7 protein amount of IL-17 was elevated.

Contrary to that, IL-10 was found to be decreased in both placenta and maternal serum in PE (Borekci et al., 2007; Aggarwal et al., 2019; Valencia-Ortega et al., 2019), as well as IL-4 (Arriaga-Pizano et al., 2005; Aggarwal et al., 2019). Therefore, a disturbed TNF-α/IL-10 ratio in maternal circulation is associated with PE (Salazar Garcia et al., 2018).

The study by Vitoratos *et al.* showed that TNF-α and IL-6 remain elevated in PE women, resulting in persistent inflammatory stress up to 12–14 weeks postpartum, even after all other signs of PE have resolved (Vitoratos et al., 2010). Furthermore, Freeman *et al.* observed that even after 20 years of the index pregnancy, women who had PE still exhibited a significantly higher IL-6/IL-10 ratio (Freeman et al., 2004). Hence, PE is associated with not only short-term but also long-term changes in inflammatory status (Freeman et al., 2004; Vitoratos et al., 2010).

### 4.2 Immunomodulatory effect of HCQ

**FIGURE 3:**
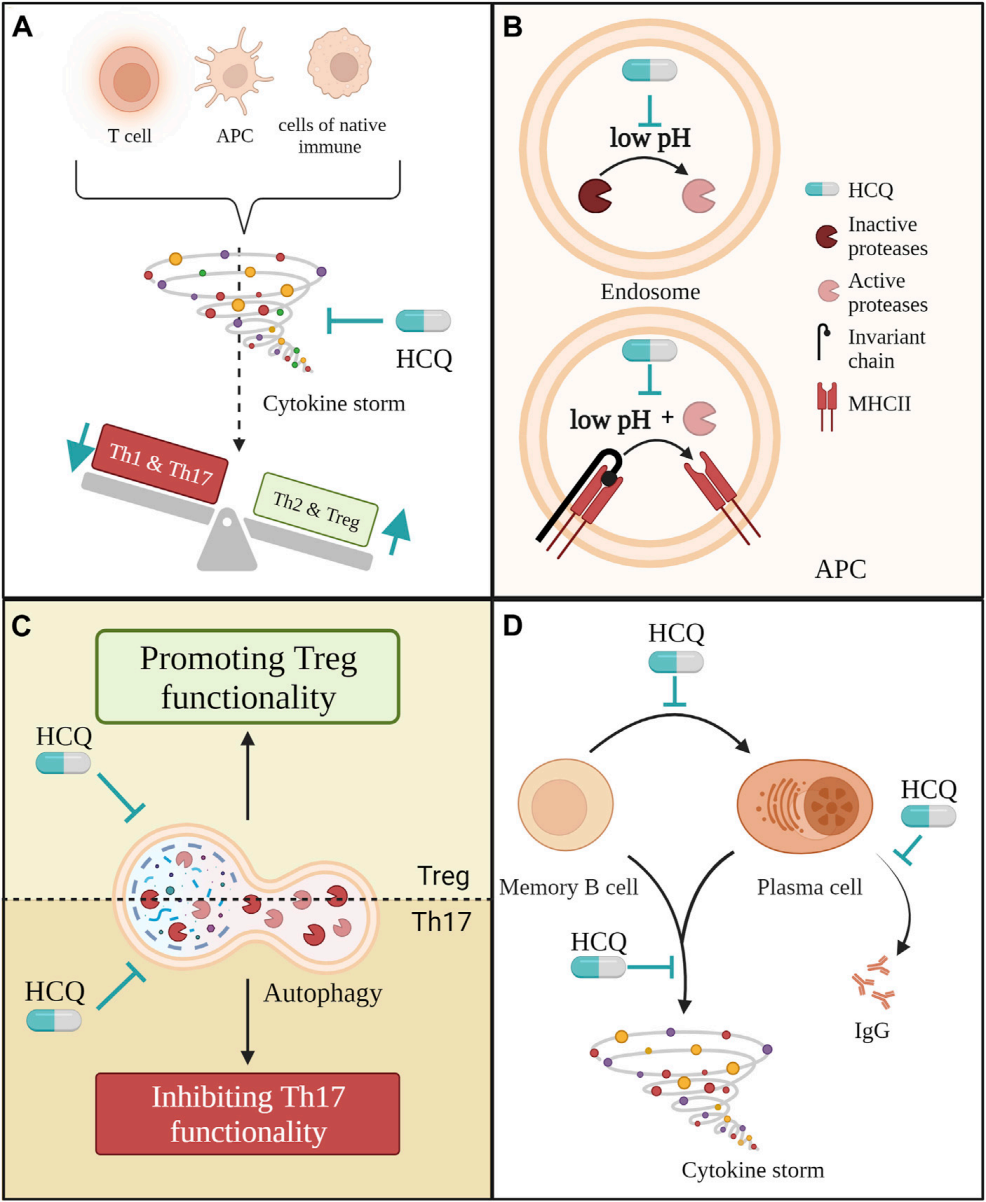
Several ways in which hydroxychloroquine (HCQ) may have an immunomodulatory effect on adaptive immune cells in PE. **(A)** HCQ could reduce cytokine production from different cell types in PE such are antigen-presenting cells, T cells and cells from native immune system like monocites, macrophages, natural killer cells and neutrophils. This way, it would reduce cytokine storm and indirectly improve the ratio between pro-inflammatory and anti-inflammatory T helper cells; **(B)** Through its direct effect on pH in endosomes HCQ could prevent the expression of MHC II molecules. Low pH is obligatory for the activation of proteases that cleave of invariant chain from MHC II as well as for the process of cleaving itself; **(C)** By affecting autophagy in Th17 and Treg cells mitigated HCQ could improve Th17/Treg ratio; **(D)** Throughout multiple different ways HCQ could reduce the pro-inflammatory effect of B cells in PE.

Although HCQ cannot directly protect cells from cytokine storms (Gajić et al., 2023), it could indirectly decrease their overall production them. We have already discussed in previous chapters how HCQ could influence cytokine production by TLRs modulation. However, that effect goes even deeper when we consider the importance of cytokine surroundings for the maturation of the adaptive immune system and its role in PE. There are two different ways in which HCQ´s immunomodulatory properties can improve PE: First, HCQ would directly inhibit TLRs activation and MHC II assembling in DCs and other antigen-presenting cells, and their cytokine production. In that way, HCQ could also affect the ratio between the Th subgroup as presented in Figure 3A. The other way is by directly affecting T and B lymphocytes.

It is a known fact that HCQ can affect the production of cytokines by different cells and consequently reduce the concentration of specific cytokines in circulation (Scharfe-Nugent et al., 2012; Scott et al., 2019; van den Borne et al., 1997; Alves et al., 2017; Piconi et al., 2011; Silva et al., 2013; Ghasemnejad-Berenji et al., 2018; Torigoe et al., 2018; Sperber et al., 1993; Ulander et al., 2021; Hjorton et al., 2018; Meng et al., 2022).

The effect of HCQ on Th1-produced cytokines was observed in SLE and HIV patients who were taking HCQ and had lower IL-6 plasma concentrations (Piconi et al., 2011). After incubation with increasing dosage of HCQ, PMBCs from SLE and RA patients reduced secretion of IL-6, IL-17 and IL-22, which are cytokines connected to Th17 cells (Silva et al., 2013). Therefore, by decreasing IL-6 production, HCQ could improve the Th17/Treg ratio (van den Borne et al., 1997; Silva et al., 2013; Sperber et al., 1993; Ulander et al., 2021) because IL-6 together with IL-1β promotes the differentiation of naive T cells into Th17 cells and even the conversion of Treg cells into Th17 cells. Since Th17 cells induce chronic inflammation, resulting in endothelial dysfunction (Saito, 2010), the HCQ effect on the Th17/Treg ratio would also have a positive effect on endothelial dysfunction. HCQ could also improve the TNF-α/IL-10 ratio in PE patients, as it has been shown in patients with repeated implantation failure (Ghasemnejad-Berenji et al., 2018). Similar to PE, these patients had high TNF-α and low IL-10 concentrations in blood circulation. However, after treatment with HCQ, the TNF-α/IL-10 ratio improved (Ghasemnejad-Berenji et al., 2018). In addition, HCQ reduces basal production of IFNs (van den Borne et al., 1997) and TLR3 and TLR9 activated (Alves et al., 2017; Hjorton et al., 2018) in PBMC from SLE patients. Moreover, it reduces the presence of endometrium in women treated with HCQ for repeated implantation failure (Ghasemnejad-Berenji et al., 2018).

Besides HCQ, CQ was also able to reduce the secretion of TNF-α, IL-6, IFN-γ and IL-1β in human primary PBMCs, monocytes/macrophages and two different monocyte cell lines stimulated with LPS or fetal DNA (Scharfe-Nugent et al., 2012; van den Borne et al., 1997; Jang et al., 2006). It has been suggested that CQ inhibited TNF-α synthesis at a post-translational step (Jang et al., 2006) while inhibiting IL-1β and IL-6 synthesis on their mRNA levels (Jang et al., 2006). In this setup, the HCQ inhibitory effect on the secretion of TNF-α, IL-6 and IL-1β was confirmed in monocytes/macrophages (Jang et al., 2006). Hypothetically HCQ should have same the effect as CQ, but this needs to be tested in an experimental setup, favourably in the context of PE. Although CQ and HCQ lead to a decrease in cytokine secretion in all Th subtypes, Th2 cytokine secretion appears to be less sensitive to their inhibitory effects compared to Th1 and Th17 cells (Schmidt et al., 2017), which would be suitable for PE treatment.

DCs are the primary and most potent antigen-presenting cells, playing an essential role in the presentation of antigens to CD8 + and CD4 + T lymphocytes and their polarization.

We have already mentioned that HCQ interferes with lysosomal acidification and TLR activation (Ponticelli and Moroni, 2017). DCs can be activated through various cell surface receptors and cytosolic sensors, but their dominant mode of activation for endogenous or exogenous nucleic acids and HMGB1 is recognized by TLR4, TLR7 and TLR9. Following activation, DCs upregulate the expression of major histocompatibility complex (MHC II) molecules, costimulatory molecules and cytokines, leading to the priming of T cells for subsequent immune responses. In SLE and RA patients, HCQ affects TLR7 and TLR9 activation and decreases the percentage of DCs as well as their production of IFN-α (Sacre et al., 2012; Alves et al., 2017; Hjorton et al., 2018; Zeidi et al., 2019; Meng et al., 2022), IL-6 (Piconi et al., 2011; Hjorton et al., 2018; Meng et al., 2022) and TNF-α (Sacre et al., 2012; Hjorton et al., 2018; Han et al., 2020).

Besides TLR activation, endosomal low pH is necessary for the activation and functioning of proteases. These enzymes are crucial for antigen presentation because they cleave the invariant chain from the MHC II molecule leaving it available for antigen. As shown in Figure 3B, by alkalisation of the lysosome, HCQ can partially inhibit the activation of proteases as well as block the cleaving of the invariant chain from MHC II. As a result, antigen presentation by antigen-presenting cells, such as DCs, is inhibited (Honey and Rudensky, 2003; Al-Bari, 2015). In both cases, by blocking the activation of DCs and by blocking MHC II assembling, HCQ would indirectly affect the Th cells ratio in PE.

Besides the indirect effect on antigen-presenting cells and cytokine storm, HCQ could also directly affect T and B lymphocytes. Another mechanism through which HCQ could affect Th17/Treg ratio is by inhibiting autophagy in both cell populations. In experiments with rodents and PBMC isolated from SLE and HIV patients, HCQ attenuated Th17 differentiation, proliferation, and production of IL-17 while promoting the immunosuppressive effect of Tregs (Piconi et al., 2011; An et al., 2017; Yang et al., 2018; Kowatsch et al., 2023). Due to that, An *et al.* suggested that HCQ might have different effects of autophagy on diverse T-cell subsets (An et al., 2017). This difference in effect on autophagy in Th17 and Treg is presented in Figure 3C. While HCQ reduces IL-17 secretion by inhibiting autophagy in Th17 cells, thereby also reversing Th17 cell over-activity, it leads to blockage of autophagy-induced degradation of Foxp3 in Treg cells, thereby promoting Treg cell functionality (An et al., 2017).

Besides priming T cells, HCQ also affects their proliferation. Both CQ and HCQ have been found to suppress T-cell proliferation (Piconi et al., 2011; Kim et al., 2021; Hu Y. et al., 2022). The suggested mechanism behind it is that HCQ causes an increase in O_2_^−^ (superoxide) production during T-cell receptor stimulation, which leads to the inhibition of T-cell proliferation (Kim et al., 2021).

Further, HCQ also affects B lymphocytes as shown in Figure 3D. HCQ leads to a decrease of TNF-α, IL-6 and IL-1β secretion by all subsets of B cells (Torigoe et al., 2018). Notably, the percentage of memory B cells and plasma cell precursors is significantly increased in PE women (Liao et al., 2009). Furthermore, the percentages of plasma cells generated upon *in vitro* stimulation with CpG were significantly higher in the PE group than in the control group (Liao et al., 2009). These cells present another potential target for HCQ since HCQ effectively suppresses the class-switch of memory B cells to plasma cells and also decreases the production of IgG (Torigoe et al., 2018; Kowatsch et al., 2023), both of which are increased in PE (Liao et al., 2009).

## 5 Vascularity in PE and HCQ

In a typical placental implantation process, the extravillous trophoblast migrates into the maternal uterine vasculature, resulting in significant alterations to the maternal spiral arteries, which become high-capacity, high-flow vessels (Rana et al., 2019). However, in pregnancies with subsequent PE, there are abnormalities in extravillous trophoblast behaviour that contribute to shallow placental implantation, leading to incomplete spiral artery transformation and placental ischemia (Rana et al., 2019). This will affect the vasculature of both mother and foetus, leading to high blood pressure and organ failure in mothers and potential fetal growth restriction (Thilaganathan and Kalafat, 2019; Opichka et al., 2021).

### 5.1 Angiogenic factors and endothelial dysfunction in PE

PE is a complex disorder characterized by an imbalance of pro- and anti-angiogenic factors, which directly influence endothelial function. Angiogenesis and the importance of nitric oxide (NO) for health and PE have been reviewed in detail in the following papers (Sutton et al., 2020; Opichka et al., 2021). Here we will focus on the most important molecules and processes to explain the potential effect of HCQ on this part of PE (Sutton et al., 2020; Opichka et al., 2021).

The equilibrium between circulating pro-angiogenic and anti-angiogenic factors has been implicated as a mechanism underlying endothelial and NO dysfunction in PE (Opichka et al., 2021). The main players in this interplay are VEGFA, PlGF, sFLT1, sENG and endothelin 1 (ET1). In healthy pregnancies, the pro-angiogenic factors, VEGFA and PlGF, bind to VEGF1 and VEGF2 receptors, stimulating angiogenesis, vascular permeability, and cell migration (Geva et al., 2002; Chau et al., 2017). These factors also lead to robust activation of endothelial nitric oxide synthase (eNOS) (Papapetropoulos et al., 1997; Grummer et al., 2009), increasing the availability of NO and promoting healthy vascular function. However, in PE, both VEGFA and PlGF are significantly decreased, contributing to the endothelial dysfunction observed in this condition (Molvarec et al., 2011; Molvarec et al., 2015; Chau et al., 2017; He et al., 2018).

The soluble form of Vascular Endothelial Growing Factor Receptor 1 (VEGFR1), also known as sFLT-1, is found to be increased in PE (Molvarec et al., 2011; Molvarec et al., 2015; He et al., 2018; Ozeki et al., 2019). This soluble receptor acts as a scavenger for VEGFA, further reducing its bioavailability in maternal serum. It has been shown that sFlt-1 secretion is increased in HTR-8/SVneo trophoblast cell line, pregnant mice and term placenta explants when challenged with either TLR3, TLR4 or TLR9 agonists (Nakada et al., 2009; He et al., 2018; Scott et al., 2019). The sFlt-1/PlGF ratio is found to be disturbed in PE, its utility as a predictive and even diagnostic biomarker has been described by various authors (Molvarec et al., 2011; Verlohren et al., 2012; Molvarec et al., 2015; Herraiz et al., 2018). Besides in patients, the difference in sFlt-1/PlGF was also observed in the secretion profile of villus tissue explants from term placentas incubated with TLR9 agonist. In addition to VEGF-related factors, sENG has been found to be elevated in PE (Ozeki et al., 2019) and is also considered a potential diagnostic tool. In addition, when challenged with PE sera JEG-3 and Sw.71 cells, a model for placental trophoblast, increased secretion of ENG (Aoki et al., 2012; Ozeki et al., 2019). Another potent vasoconstrictor that incites reactive oxygen species production and inflammatory signalling in endothelial cells is ET1 (Kowalczyk et al., 2015).

Endothelial dysfunction is one key feature of PE (Chambers et al., 2001; Echeverria et al., 2020), manifesting a barrier disruption and impaired vasodilatory capacity. Studies have demonstrated that NO availability is diminished in women with PE (Sutton et al., 2020). Oxidative stress and elevated levels of ROS are commonly observed in PE and likely contribute to impaired NO signalling and vascular dysfunction (McElwain et al., 2020). Furthermore, this leads to overall vasocontraction and high blood pressure. Therefore, affecting any of these molecules would be beneficial for PE treatment.

#### 5.1.1 HCQ impact role on angiogenic factors and endothelial dysfunction

Data concerning the effect of HCQ on angiogenic molecules and endothelial dysfunction appears to be quite heterogeneous. Concerning angiogenic molecules, HCQ seems to reduce sFLT-1 secretion from cytotrophoblast cells and slightly increase PlGF, but it does not seem to affect ENG or the secretion of these molecules from placental explants (Scott et al., 2019; Rahman et al., 2020; Kadife et al., 2022). However, HCQ reversed the effect of TLR9 agonist on third-trimester placenta explants secretion, leading to a reduction in sFlt-1 production and improvement of sFlt1/PlGF ratio (Scott et al., 2019).

**FIGURE 4:**
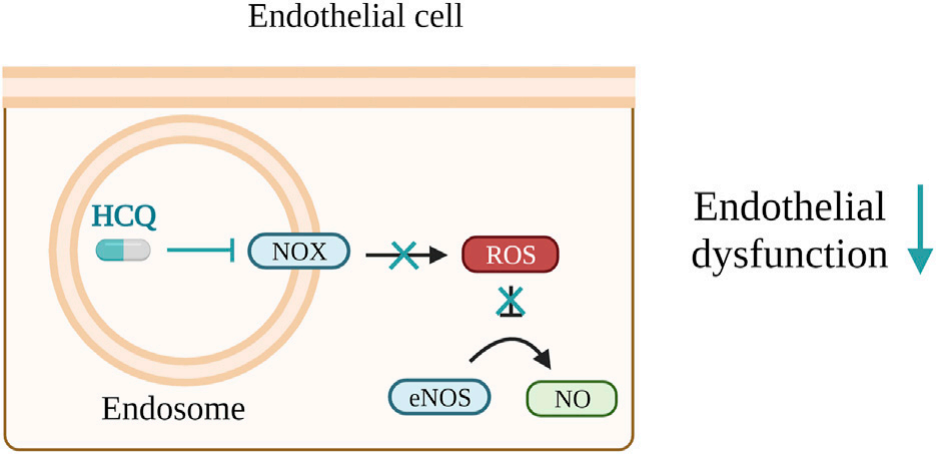
Hydroxychloroquine (HCQ) lowers reactive oxygen species (ROS) levels, enhances nitrogen monoxide (NO) bioavailability, and contributes to the prevention of endothelial dysfunction. These effects are accomplished by either inhibiting the activity of NADPH oxidase (NOX) within endosomes or by regulating endothelial nitric oxide synthase (eNOS).

In a mouse model for SLE and APS, HCQ reduces ROS levels, increases NO bioavailability and helps to prevent endothelial dysfunction. This is achieved either through the blocking of NADPH oxidase (NOX) present in endosomes or by modulation of eNOS, respectively (Gomez-Guzman et al., 2014; Miranda et al., 2019). The same mechanism presented in Figure 4 would be also beneficial for PE patients. Consistent with this, HCQ demonstrated a reduction in clot formation and thrombin generation in adult endothelial cells, APS mouse model and patients with primary APS (Nuri et al., 2017; Miranda et al., 2019; Kravvariti et al., 2020). Since thrombin generation is elevated in PE (de Boer et al., 1989; Xin et al., 2012) use of HCQ would be beneficial in this case, too. In the context of PE, HCQ has been shown to reduce PE serum-triggered production of ET-1 and NOX in HUVECs (Rahman et al., 2016). However, conflicting results have been obtained on the same type of cells for TNF-α-triggered ET-1 secretion (Rahman et al., 2016; Kadife et al., 2022).

Furthermore, HCQ seems to have opposing effects on fetal endothelial cells under different conditions. For instance, in high-glucose conditions, HCQ decreased ROS production in endothelial cells but also diminished NO production and angiogenesis (Rezabakhsh et al., 2017). Conversely, when exposed to a mixture of pro-inflammatory cytokines, HCQ did not affect angiogenesis in fetal arterial endothelial cells (Gajić et al., 2023), while in the presence of just TNF-α, HCQ improved angiogenesis in HUVECs (Rahman et al., 2016).

The high variability of the HCQ effect on angiogenic factors and endothelial dysfunction is due to differences in tested conditions. Thus, these special effects of HCQ need to be confirmed in PE or PE-like experimental conditions.

### 5.2 HCQ and regulators of leukocyte rolling and adhesion

An alternative mechanism, by which HCQ might affect PE is its effect on the regulation of leukocyte rolling and adhesion. The most important molecules in this process are ICAM-1, VCAM-1 and selectins (McEver and Zhu, 2010). When endothelial cells undergo inflammatory activation, they increase the expression of selectins, VCAM-1, and ICAM-1. In turn, they promote monocytes adherence and transfer to sites of inflammation.

Circulating soluble forms of all three molecules (ICAM-1, VCAM-1 and Selectin E and P) were found to be increased in PE (Austgulen et al., 1997; Kim et al., 2004; Molvarec et al., 2011; Dogan et al., 2014; Docheva et al., 2019; Valencia-Ortega et al., 2019; Kornacki et al., 2020). Furthermore, sVCAM was specifically more prominent in severe and EO-PE (Kim et al., 2004; Dogan et al., 2014). At the same time, multiple authors even found a correlation between sVCAM-1 and sICAM-1 with the systolic/diastolic blood pressure values, and renal and liver function parameters in PE patients (Szarka et al., 2010; Dogan et al., 2014).

It has been shown that HCQ induces a decline of VCAM-1 in endothelial cells (Le et al., 2014; Li et al., 2018; Hu Y. et al., 2022; Kadife et al., 2022; Gajić et al., 2023). It has been suggested that this inhibition goes through the activation of ERK5 (Le et al., 2014; Cheng et al., 2018). Alongside the reduction of VCAM-1, multiple studies have reported a decrease in ICAM-1 (Li et al., 2018; Sciascia et al., 2020; Hu Y. et al., 2022). Furthermore, transcription of Selectin P was negatively correlated with HCQ dose in platelets from SLE patients and this confirmed HCQ (Cornwell et al., 2021). Furthermore, there is an association between high HCQ blood levels and reduced thrombotic events in SLE patients (Petri et al., 2021).

Besides the effect on gene and protein levels, HCQ was also able to decrease the serum concentrations of sICAM, sVCAM and sSELE in insulin resistance-induced endothelial dysfunction in rats (Abdel-Hamid and Firgany, 2016). Further, HCQ improves altered endothelium-dependent relaxation in the context of APS (Miranda et al., 2019). Therefore, HCQ could potentially decrease the concentration of circulating VCAM-1, ICAM-1 and selectins in PE patients, which potentially could affect their high blood pressure. Moreover, HCQ could improve endothelial-dependent relaxation and decrease thrombosis in PE patients. Besides the effect on the vasculature, by reducing VCAM-1 on endothelial cells, HCQ could also decrease monocyte penetration to tissue and inflammatory reactions in general.

## 6 Conclusion

While there is a substantial body of literature discussing the potential utility of HCQ as a treatment or prophylaxis for PE, the current state of research falls short in terms of rigorous experimental and clinical investigation in the context of PE. The majority of available studies draw upon HCQ data derived from patients with conditions such as SLE and RA, leaving a noticeable gap in dedicated research on PE. Furthermore, some clinical studies reporting a reduced incidence of PE in pregnant women who used HCQ are essentially meta-analyses of trials conducted with SLE patients.

Given that the effectiveness of HCQ appears to be influenced by the specific disease or condition for which it is being tested, it is imperative to explore its impact under PE-specific conditions. In our review, we endeavoured to elucidate the mechanisms of HCQ action and identify areas of convergence with the pathophysiology of PE. More precisely, we aimed to highlight distinct molecular targets, cell types, and potential outcome measures that could guide future experimental investigations.
